# Growth, Nutrient Retention, Waste Output, and Antioxidant Capacity of Juvenile Triangular Bream (*Megalobrama terminalis*) in Response to Dietary Selenium Yeast Concentration

**DOI:** 10.1155/2022/9242188

**Published:** 2022-09-22

**Authors:** Yubo Wu, Hengjia Ma, Donghuan Fu, Hui Zhu, Xiujuan Wang, Xing Ren

**Affiliations:** ^1^College of Life Sciences, Taizhou University, Taizhou, 318000 Zhejiang, China; ^2^Hangzhou Academy of Agricultural Sciences, Hangzhou, 310024 Zhejiang, China; ^3^Key Laboratory of Tropical Marine Ecosystem and Bioresource, Guangxi Key Laboratory of Beibu Gulf Marine Resources, Environment and Sustainable Development, Forth Institute of Oceanography, Ministry of Natural Resources, Beihai, 536000 Guangxi, China

## Abstract

An 8-week feeding trial was conducted to investigate the effects of different dietary selenium yeast levels on growth, nutrient retention, waste output, and antioxidant capacity of juvenile triangular bream (*Megalobrama terminalis*). Five isonitrogenous (320 g/kg crude protein) and isolipidic (65 g/kg crude lipid) diets were formulated, with supplementation of graded levels of selenium yeast at 0 (diet Se0), 1 (diet Se1), 3 (diet Se3), 9 (diet Se9), and 12 g/kg (diet Se12). No significant differences were found in initial body weight, condition factor, visceral somatic index, hepatosomatic index, and whole body contents of crude protein, ash, and phosphorus among fish fed different test diet. The highest final body weight and weight gain rate were found in fish fed diet Se3. The specific growth rate (SGR) is closely related to dietary selenium (Se) concentrations with a relationship described as SGR = –0.0043 Se^2^ + 0.1062 Se + 2.661. Higher feed conversion ratio was found, while lower retention efficiencies of nitrogen and phosphorus were found in fish fed diets Se1, Se3, and Se9 than in fish fed diet Se12. Contents of selenium in whole body, vertebra, and dorsal muscle increased with dietary supplementation of selenium yeast increased from 1 mg/kg to 9 mg/kg. Lower nitrogen and phosphorous waste was found in fish fed diets Se0, Se1, Se3, and Se9 than in fish fed diet Se12. Fish fed diet Se3 exhibited the highest activities of superoxide dismutase, glutathione peroxidase, and lysozyme while the lowest malonaldehyde content in both the liver and kidney. Our results showed that the optimal dietary selenium requirement for triangular bream should be 12.34 mg/kg based on the nonlinear regression on SGR, and fish fed diet Se3 in which selenium concentration (8.24 mg/kg) was close to the optimal requirement displayed the best growth performance, feed nutrient utilization, and antioxidant capacity.

## 1. Introduction

Minerals are essential to the growth and development of fish and must be obtained through diet since fish itself cannot synthesize them [1]. As one essential mineral for fish, selenium (Se) is vital for normal growth, physiological functions, and maintenance of tissue homoeostasis at trace amounts [2], and its deficiency has been demonstrated to exert various negative effects such as suppression of growth performance, decline of feed utilization and immunity, increase of oxidative stress, and mortality in fish [3–6]. Studies indicated that tolerance of selenium in fish is in a narrow range, and dietary selenium requirements exhibit diversity among various species, ranging 0.79–0.81 mg Se/kg diet for cobia (*Rachycentron canadum*) [7], 1.06–2.06 mg Se/kg diet for Nile tilapia (*Oreochromis niloticus*) [8], and 4 mg Se/kg diet for meagre (*Argyrosomus regius*) [9], which means it is infeasible to extrapolate dietary selenium supplemental amount from one species to another. Therefore, it is necessary to investigate the optimal dietary supplementation of selenium for a specific fish species.

Studies suggest that supplementation of organic selenium (selenomethionine) rather than its inorganic form (Na_2_SeO_3_) generates greater absorption and retention rate in fish [10–12]. Selenium yeast contains predominantly selenomethionine and a handful of other Se-containing compounds [13] and has been reported to enhance growth performance, feed utilization, and immunocompetence in numerous fish species such as Wuchang bream (*Megalobrama amblycephala*) [14], hybrid striped bass (*Morone chrysops* × *Morone saxatilis*) [15], and rainbow trout (*Oncorhynchus mykiss*) [16].

Triangular bream (*Megalobrama terminalis*) is a freshwater omnivorous fish species with high value and is extensively farmed in China because of its rapid growth, delicious taste, and strong disease resistance [17]. Until now, nutritional studies on triangular bream are almost blank except for that dietary protein requirement has been determined [18], and meanwhile, dietary requirement for selenium of this fish species is unknown although selenium is essential for its growth and development. Therefore, the present study utilizes selenium yeast as the selenium source, aiming to evaluate the effects of different selenium yeast levels on growth, feed utilization, body composition, waste output, and antioxidant response of triangular bream and meanwhile determine the dietary selenium requirement of this fish species.

## 2. Materials and Methods

### 2.1. Feed Ingredients and Test Diets

Steam dried red fishmeal, rapeseed meal, cottonseed meal, poultry by-product meal, rice bran, wheat flour, zeolite powder, calcium hydrophosphate, choline chloride, and vitamin premix were supplied by Zhejiang Hongli Feed Stock Co., Ltd. (Huzhou, China). Brewer's dried yeast and selenium yeast (1600 mg/kg Se content) were purchased from Angel Yeast Co., Ltd. (Yichang, China), and selenium free mineral premix was offered by Huzhou Younengte Biotechnology Co., Ltd. (Huzhou, China).

Five isonitrogenous, isolipidic, and isoenergetic diets were designed with supplementation of selenium yeast at 0, 1, 3, 9, and 12 g/kg and were correspondingly abbreviated as diets Se0, Se1, Se3, Se9, and Se12, which provided the actual dietary selenium concentration of 1.46, 4.10, 8.24, 22.87, and 29.96 mg/kg, respectively. All the test diets were formulated to contain 320 g/kg crude protein and 65 g/kg crude lipid. Formulation, proximate composition, and selenium content of the test diets are shown in Table 1.

All feed stuffs were smashed with a Chinese herbal medicine grinder and filtered through a 50-mesh sieve. Each of the feed ingredients was weighed according to feed formula and then mixed thoroughly with a blending machine (Shenzhen Jinjie Industrial Equipment Co., Ltd.) to ensure homogenous blends, and finally, fish oil were added and fully rubbed. 150 g/kg water was added to the above mixture, stirred for 10 min, and subsequently pelletized into 2.0 mm diameter pellets by a pelletizer (Hebei Xingtai Giant Machinery Factory). The pellets were dried in laboratory oven at 50°C, packed in Ziploc bag, and then stored in -20°C until use.

### 2.2. Fish and Husbandry Management

The feeding trial was carried out at the aquaculture base of Hangzhou Academy of Agricultural Sciences (Hangzhou, China). Triangular bream juveniles were purchased from a freshwater fish hatchery in Deqing county (Huzhou, China). Upon arrival, the juvenile fish were domesticated in a square cement tank (7 m × 6 m × 1.6 m) and fed diet Se0 for two weeks.

At the end of adaptive period, the acclimatized juvenile fish were deprived of feed for 24 h and pooled. Fifteen groups of 40 fish each were group-weighed and randomly distributed into 15 experimental net pen cages (1 m × 1 m × 1.5 m), with each test diet triplicated. Initial body weight of fish was 4.26 ± 0.3 g (mean ± SD, *n* = 15). Three groups of 20 fish each were collected and stored at -20°C for the analysis of initial body composition.

Experimental fish were fed twice daily (08 : 30 h and 16 : 00 h) to apparent satiation during the eight-week feeding trail. Dead fish were collected and weighed for the correction calculation of feed conversion ratio (FCR). 30% water was released, and then, new water was added into the net pen every week. Water temperatures were recorded daily and ranged 26.7-30.2°C. Dissolved oxygen (>7.0 mg/L) and ammonia nitrogen (<0.1 mg/L) were monitored in the laboratory of Fisheries Research Institution of Hangzhou Academy of Agriculture Science every two weeks.

At the close of the feeding trial, fish were deprived of feed for 24 h, captured from each net cage, anesthetized with 50 mg/L MS-222 (Sigma-Aldrich), and finally group-weighed. Ten fish from each net cage were randomly collected for the analysis of final body composition. Another three fish from each net cage were randomly selected to collect the liver and kidney samples. After anatomy, dorsal muscle and vertebra samples were collected. All the samples were stored at -80°C until use.

### 2.3. Chemical Analysis

The sampled fish were weighed and then autoclaved at 120°C for 20 min with high temperature sterilizing oven (Shanghai Shenan Medical Instrument Factory), homogenized with a grinder, and then dried at 105°C. The methods testing contents of moisture, crude protein, crude lipid, ash, and phosphorus in test diets and fish were analyzed using the method described in AOAC [19].

Selenium contents in test diet, fish body, dorsal muscle, and vertebra were assayed with inductively coupled plasma mass spectrometry (ICP-MS, Agilent 7900, USA) after microwave digestion. Briefly, the crushed sample was weighed and put into a 50 mL Teflon digestive tube, and 10 mL pure concentrated nitric acid was added. The sample was digested at 180°C for 30 min in a microwave digestion machine (CEM-MARS_5 XPRESS). After cooling, remove and transfer to a 50 mL colorimetric tube, wash the digestion tube twice with 20 mL ultrapure water, combine the solution, constant volume to 50 mL with 5% superior pure nitric acid, and then, test in ICP-MS after appropriate dilution.

Prior to the assay of malonaldehyde (MDA) and enzyme activity, liver and kidney samples were homogenized in ice-cold 0.86% physiological saline (1 : 4 *w*/*v*). Each homogenate was centrifuged at 12000 rpm under 4°C for 5 min, and the supernatant was isolated and used as crude extracts for assay. Concentration of the soluble protein in the supernatant was determined using the method described in Bradford [20], with the bovine serum albumin as a standard. Activities of superoxide dismutase (SOD), glutathione peroxidase (GSH-Px), and lysozyme (LZM), as well as content of MDA in the liver and kidney, were assayed using the commercial kits manufactured by Nanjing Jiancheng Bioengineering Institute (Nanjing, China) according to the manufacturer's instructions and analyzed by a standard enzyme reader (Multiskan FC, Thermo Scientific, Braunschweig, Germany).

### 2.4. Calculations

Feed intake, weight gain rate (WGR), specific growth rate (SGR), feed conversion ratio (FCR), nitrogen retention efficiency (NRE), phosphorus retention efficiency (PRE), condition factor, visceral somatic index (VSI), hepatosomatic index (HSI), nitrogen waste (NW), and phosphorus waste (PW) were calculated as that described in Wu et al. [21].

### 2.5. Statistics

All of the data were checked for normal distribution by one-sample Kolmogorov-Smirnov test and homogeneity of variances by Levene's test. The differences in the trial parameters among fish fed different test diets were analyzed with one-way ANOVA. Multiple comparisons utilizing Tukey's test were performed on the variables if significant differences were detected. Analysis of the experimental data was performed using software IBM SPSS (version 26.0, IBM Corp., Armonk, New York), and *P* < 0.05 was considered statistically significant. Moreover, the relationship between SGR and dietary selenium concentrations was examined with nonlinear regression.

## 3. Results

### 3.1. Survival, Feed Intake, Growth, and Feed Utilization

**Figure 1:**
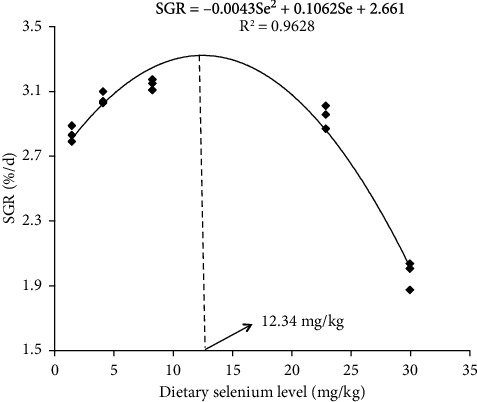
Relationship between specific growth rate (SGR) and dietary selenium (Se) concentrations for juvenile triangular bream (*Megalobrama terminalis*) fed the test diets for 8 weeks. The regression equation was described as SGR = –0.0043 Se^2^ + 0.1062 Se + 2.661 (*r*^2^ = 0.9628, *n* = 3, *P* < 0.05).

Survival was over 90% during the feeding trial. No significant difference was found in IBW among fish fed different test diets (one-way ANOVA, *P* > 0.05, Table 2). The FBW and WGR of fish climbed to peak when fed at diet Se3 and then decreased with the increase of supplementation of selenium (Tukey's test, *P* < 0.05). The SGR was closely correlated with dietary selenium content, and the regression equation was SGR = –0.0043 Se^2^ + 0.1062 Se + 2.661 (*r*^2^ = 0.9628, *n* = 3, *P* < 0.05, Figure 1). Significant lower feed intake and FCR, while higher NRE and PRE, were found in fish fed diets Se0, Se1, Se3, and Se9 than in fish fed diet Se12 (Tukey's test, *P* < 0.05, Table 2).

### 3.2. Body Composition and Selenium Content

At harvest, no significant differences were found in condition factor, VSI, and HSI, as well as whole body contents of crude protein, ash, and phosphorus (one-way ANOVA, *P* > 0.05, Table 3). The lowest and highest whole body moisture content was, respectively, found in fish fed diet Se1 and Se12 (Tukey's test, *P* < 0.05), while no significant difference was found in whole body moisture content among fish fed diets Se0, Se3, and Se9 (Tukey's test, *P* > 0.05). The lowest and highest whole body moisture content was found in fish fed diet Se12, and Se1, respectively (Tukey's test, *P* < 0.05). Contents of selenium in whole body and dorsal muscle increased with dietary selenium increase from 4.1 mg/kg to 29.96 mg/kg, while content of selenium in vertebra climbed to plateau when dietary selenium concentration reached 22.87 mg/kg (Tukey's test, *P* < 0.05, Table 4).

### 3.3. Outputs of Nitrogen and Phosphorous Wastes

**Figure 2:**
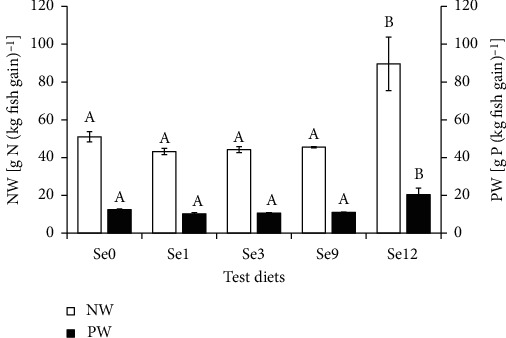
Outputs of nitrogen waste (NW) and phosphorus waste (PW) from aquaculture of juvenile triangular bream (*Megalobrama terminalis*) that fed the test diets for 8 weeks. Data were expressed as mean ± SD (*n* = 3), the columns represent mean value, and the error bars represent SD. Date with different letters are significantly different at *P* < 0.05.

Both the nitrogen and phosphorous wastes were significantly lower in fish fed diets Se0, Se1, Se3, and Se9 than in fish fed diet Se12 (Tukey's test, *P* < 0.05, Figure 2).

### 3.4. The SOD, GSH-Px, and Lysozyme Activities and MAD Content in the Liver and Kidney

Compared with fish fed diet Se0, higher hepatic SOD activity was found in fish fed diets Se1 and Se3, whereas lower hepatic SOD activity was found in fish fed diet Se12 (Tukey's test, *P* < 0.05, Table 5). In the kidney, higher SOD activity was found in fish fed diets Se1 and Se3 than in fish fed diets Se0, Se9, and Se12 (Tukey's test, *P* < 0.05). Higher hepatic GSH-Px activity was found in fish fed diets Se1, Se3, and Se9 than in fish fed diets Se0 and Se12 (Tukey's test, *P* < 0.05). Compared with fish fed diet Se0, higher activity of GSH-Px in the kidney was found in fish fed diets Se1 and Se3, and lower activity of GSH-Px in the kidney was found in fish fed diets Se9 and Se12 (Tukey's test, *P* < 0.05). The highest hepatic lysozyme activity was found in fish fed diets Se3 and Se9, followed by that in fish fed diets Se0 and Se1, and the lowest was found in fish fed diet Se12 (Tukey's test, *P* < 0.05). In the kidney, the highest lysozyme activity was found in fish fed diet Se3, and the lowest was found in fish fed diets Se9 and Se12 (Tukey's test, *P* < 0.05). When fish were fed with diets Se1 and Se3, lower MDA content was found in both the liver and kidney compared with fish fed with other test diets (Tukey's test, *P* < 0.05).

## 4. Discussions

Early studies indicated that a numerous freshwater fish species, such as Nile tilapia (*Oreochromis niloticus*) [8], common carp (*Cyprinus carpio*) [22], largemouth bass (*Micropterus salmoides*) [23], and gibel carp (*Carassius auratus gibelio*) [24], have a requirement for selenium, and it is speculated that dietary supplementation of selenium maybe also necessary for triangular bream. Therefore, in the present study, five graded levels of selenium yeast were added in the test diets to determine the optimal requirement for selenium of triangular bream, and our results showed that in comparison with fish fed diet Se0, higher FBW and WGR were found in fish fed diets Se1 and Se3, whereas lower FBW and WGR were found in fish fed diet Se12, and meanwhile, fish fed diet Se3 showed the highest FBW and WGR, suggesting that dietary selenium concentration threshold for triangular bream should not be exceed that determined in diet Se12 (29.96 mg Se/kg diet), and among the test selenium levels, the maximum growth capacity of this fish species is achieved when fed diet Se3 (8.24 mg Se/kg diet). Based on the nonlinear regression analysis on SGR, the optimal dietary selenium requirement for the fast growth of triangular bream should be 12.34 mg/kg, which was much higher than that determined for any fish species reported until now. For instance, the optimum dietary requirement of selenium sourced from selenomethionine was 0.79 mg/kg for grouper (*Epinephelus malabaricus*) [6] and cobia (*Rachycentron canadum*) [7], according to the regression analysis on weight gain and SGR, respectively. Dietary selenium concentration around 1 mg/kg can sustain the fastest growth of Nile tilapia (1.06 mg/kg, [8]) and gibel carp (*Carassius auratus gibelio*) (1.18 mg/kg, [24]). Utilizing Na_2_SeO_3_ as a selenium source, the optimum dietary selenium requirement for largemouth bass (*Micropterus salmoides*) based on weight gain was 1.60 mg/kg [23]. Le and Fotedar [25] reported that the optimum dietary selenium requirement for yellowtail kingfish (*Seriola lalandi*) based on weight gain was 5.56 mg/kg, with Se-yeast served as the selenium source. The requirement of selenium for cutthroat trout (*Oncorhynchus clarkii*) is 9.2 mg/kg with selenomethionine as selenium source [26]. The differences that exist between the above-mentioned studies may ascribe to the difference on fish species, dietary selenium source, or even the evaluation standard.

As reported in studies conducted on coho salmon (*Oncorhynchus kisutch*) [27], meagre [9], black sea bream (*Acanthopagrus schlegelii*) [28], Nile tilapia [8], cobia [7], and common carp [29], selenium generally acts as a promoter to improve nutrients deposition via the enhancement of feed utilization efficiency in fishes that were fed at suitable dietary selenium concentrations. In the present study, higher NRE and PRE were found, whereas relative lower FCR was found in fish fed diets Se1, Se3, and Se9 than in fish fed diet Se12, which suggested that dietary inclusion of selenium that ranges 4.10-22.87 mg/kg is beneficial for ameliorating feed utilization efficiency in triangular bream. Furthermore, our results showed that the waste output of nitrogen and phosphorus in the aquaculture of triangular bream was not influenced when feeding at suitable dietary selenium concentration (4.10-22.87 mg/kg), whereas would be greatly increased with dietary selenium included superfluously.

Somatic indices like condition factor, visceral somatic index (VSI), and hepatosomatic index (HSI) are crude measures for both nutritional and healthy status of fish [30]. In the present study, no significant statistically differences were found in condition factor, VSI, and HSI among fish fed different test diets, indicating that both nutritional and healthy status of triangular bream were not affected by dietary inclusion levels of selenium. Similar results were also reported in early researches. For instance, Du et al. [27] reported that condition factor, HSI, and intestinal somatic index in coho salmon showed no statistically differences when fed at various dietary selenium levels. The condition factor and VSI in largemouth bass were not influenced by dietary selenium concentrations [23]. Condition factor and HSI in gibel carp [23, 24] and black sea bream [28] showed no response to dietary selenium levels.

The previous studies indicated that dietary selenium treatment did not affect whole body proximate composition of some fish species such as crucian carp (*Carassius auratus gibelio*) [31], largemouth bass [23], common carp [22], and yellowtail kingfish [10]. In the present study, compared with fish fed diet Se0, no significant statistically differences were found in whole body contents of moisture, crude protein, ash, and phosphorous among fish fed the other four test diets, which implied that the whole body components of triangular bream were not affected by dietary inclusion levels of selenium.

Selenium modulates bone metabolic turnover in favor of bone formation [32], which is associated with its function on improving growth. Thus, bony selenium saturation is a reliable indicator to determine dietary selenium requirement for fast-growing fish. In the present study, vertebral selenium content increased with dietary selenium increased from 0 to 22.87 mg/kg and then reached to a plateau of deposition with dietary selenium further increased. This result indicated that, with vertebral selenium saturation as an evaluation criterion, the optimal supplementation level of selenium yeast in diet for triangular bream was in consistent with the results evaluated based on the criterions of growth performance and feed utilization. Different from vertebra, contents of selenium in whole body and dorsal muscle of triangular bream increased in a dose-dependent manner. Similarly, selenium concentrations in the liver of grouper [6]; in the liver and whole body of cobia [7]; in the hepatopancreas, muscle, and whole body of gibel carp [24]; and in the liver, muscle, and gill of Nile tilapia [8] also have been observed to increase with increasing dietary selenium levels.

MDA, one product of lipid peroxidation, is frequently measured as a marker of oxidative stress in organisms [33]. In the current study, the MDA concentration in the liver and kidney of fish decreased firstly with dietary selenium level increased form 1.46 mg/kg to 8.24 mg/kg and then increased when dietary selenium content further increased, which implied that triangular bream fed diet Se3 generated the least oxidative stress. Similar results were found in coho salmon [27], in which hepatic MDA content dropped firstly and then increased with dietary selenium continually increased. Inversely, MDA content in serum of black sea bream tended to decrease with the increase of dietary selenium concentration [28]. Zhu et al. [23] also reported that hepatic MDA concentration in largemouth bass is not affected by dietary selenium levels. The differences on fish species, test tissues, or even cultural conditions may explain the various results among different investigations.

Selenium plays a vital role in maintaining the activities of numerous selenoproteins, especially Se-dependent glutathione peroxidase [23, 24]. In our study, activities of GSH-Px, SOD, and lysozyme in both the liver and kidney were significantly improved when fish were fed diets supplemented with 1.46–8.24 mg Se/kg, which indicated that appropriate dietary selenium effectively increased oxidative resistance and nonspecific immunity of triangular bream. Similar responses were reported for activities of GSH-Px, SOD, and lysozyme in the liver of coho salmon [27], cobia [7], and grouper [6], as well as in serum of black sea bream [28], with dietary selenium content increased within the optimal requirement level.

To conclude, triangular bream feeding diet Se3 grows best, and the optimal dietary selenium requirement for its fast growth should be 12.34 mg/kg, based on the nonlinear regression analysis on SGR. Increasing dietary selenium inclusion within the optimal requirement level, the feed utilization efficiency (FCR and NRE), antioxidant capacity, and nonspecific immunity activity of triangular bream were enhanced, and whole body components and waste output of nitrogen and phosphorus were not influenced.

## Figures and Tables

**Table 1:** Formulation (g/kg), proximate composition (g/kg), contents of energy (MJ/kg), and selenium (mg/kg) in the test diets.

Ingredients	Se0	Se1	Se3	Se9	Se12
Steam dried red fishmeal	70	70	70	70	70
Soybean meal	240	240	240	240	240
Rapeseed meal	90	90	90	90	90
Cottonseed meal	70	70	70	70	70
Poultry by-product meal	50	50	50	50	50
Rice bran	160	160	160	160	160
Wheat flour	200	200	200	200	200
Zeolite powder	20	20	20	20	20
Calcium hydrophosphate	20	20	20	20	20
Vitamin premix	7	7	7	7	7
Se free mineral premix	3	3	3	3	3
Brewer's dried yeast	30	29	27	21	18
Selenium yeast	0	1	3	9	12
Fish oil	40	40	40	40	40
Total	1000	1000	1000	1000	1000
Proximate analyses					
Crude protein	324	324	324	324	324
Crude fat	67	67	67	67	67
Crude ash	73	73	73	73	73
Gross energy	17.3	17.3	17.3	17.2	17.1
Selenium content	1.46	4.10	8.24	22.87	29.96

Vitamin and mineral premix (per kg diet): thiamin, 20 mg; riboflavin, 20 mg; pyridoxine, 20 mg; cyanocobalamine, 2 mg; retinyl palmitate, 1.38 mg; calciferol, 0.5 mg; phylloquinone, 10 mg; folic acid, 5 mg; d-calcium pantothenate, 50 mg; phaseomannite, 100 mg; all-rac-*α*-tocopheryl acetate, 10 mg; mineral premix provides per kg of feed: NaCl, 0.8 mg; MgSO_4_·7H_2_O, 12 mg; NaH_2_ PO_4_·7H_2_O, 20 mg; KH_2_PO_4_, 25.6 mg; Ca(H_2_PO_4_)_2_·H_2_O, 16 mg; FeSO_4_, 2 mg; Ca(CH_2_CHCOO)_2_·5H_2_O, 2.8 mg; ZnSO_4_·7H2O, 0.028 mg; MnSO_4_·4H_2_O, 0.013 mg; CuSO_4_·5H_2_O, 0.0025 mg; CoC_l2_·6H_2_O, 0.0008 mg; KIO_3_·6H_2_O, 0.0024 mg.

**Table 2:** Initial body weight (IBW, g/fish), final body weight (FBW, g/fish), weight gain rate (WGR, %), special growth rate (SGR, %/d), feed intake, feed conversion rate (FCR), nitrogen retention efficiency (NRE, %), phosphorus retention efficiency (PRE, %), and survival rate (%) of juvenile triangular bream (*Megalobrama terminalis*) fed the test diets.

Diet	IBW	FBW	WGR	SGR	Feed intake	FCR	NRE	PRE	Survival
Se0	4.29 ± 0.04	23.51 ± 0.90^b^	448.5 ± 16.3^b^	2.84 ± 0.05^b^	2.62 ± 0.05^ab^	1.14 ± 0.03^a^	34.06 ± 1.47^b^	31.19 ± 2.44^ab^	99.2
Se1	4.26 ± 0.01	26.28 ± 0.19^cd^	517.2 ± 2.9^cd^	3.03 ± 0.01^c^	2.46 ± 0.04^a^	1.02 ± 0.03^a^	37.76 ± 0.66^b^	36.48 ± 2.19^b^	98.8
Se3	4.27 ± 0.06	28.17 ± 0.64^d^	559.3 ± 13.0^d^	3.14 ± 0.03^c^	2.54 ± 0.02^a^	1.03 ± 0.01^a^	36.81 ± 1.67^b^	35.43 ± 1.53^b^	99.2
Se9	4.23 ± 0.02	25.34 ± 0.44^bc^	499.4 ± 14.0^c^	2.98 ± 0.04^bc^	2.52 ± 0.01^a^	1.06 ± 0.01^a^	36.60 ± 0.40^b^	34.16 ± 0.56^b^	100
Se12	4.23 ± 0.04	13.82 ± 0.82^a^	226.7 ± 16.7^a^	1.97 ± 0.09^a^	2.73 ± 0.11^b^	1.68 ± 0.20^b^	21.54 ± 2.86^a^	23.80 ± 3.76^a^	90

Data are presented as mean ± SD (*n* = 3). The data with different superscripts in the same row mean significant difference at *P* < 0.05.

**Table 3:** Condition factor (g/cm^3^), visceral somatic index (VSI, %), hepatosomatic index (HSI,%), and proximate composition (%) of juvenile triangular bream (*Megalobrama terminalis*) fed the test diets for 8 weeks.

Diet	Condition factor	VSI	HSI	Moisture	Crude protein	Crude lipid	Ash	Phosphorus
Se0	1.73 ± 0.09	8.15 ± 0.73	1.22 ± 0.15	73.94 ± 0.15^ab^	16.28 ± 0.17	5.71 ± 0.07^ab^	3.26 ± 0.06	0.56 ± 0.05
Se1	1.82 ± 0.08	7.98 ± 0.59	1.15 ± 0.24	73.22 ± 0.93^a^	15.96 ± 0.5	7.07 ± 0.27^c^	3.23 ± 0.07	0.57 ± 0.02
Se3	1.85 ± 0.12	7.99 ± 0.80	1.11 ± 0.20	73.73 ± 0.68^ab^	16.02 ± 0.48	6.41 ± 0.35^bc^	3.20 ± 0.08	0.57 ± 0.02
Se9	1.76 ± 0.08	7.78 ± 1.14	1.13 ± 0.11	74.17 ± 1.02^ab^	15.96 ± 0.60	6.52 ± 0.29^c^	3.19 ± 0.12	0.55 ± 0.03
Se12	1.69 ± 0.17	8.48 ± 1.46	1.32 ± 0.30	75.69 ± 0.75^b^	15.31 ± 0.51	5.32 ± 0.27^a^	3.34 ± 0.11	0.60 ± 0.02

Data are presented as mean ± SD (*n* = 3). The data with different superscripts in the same row mean significant difference at *P* < 0.05.

**Table 4:** Selenium content (mg/kg) in different tissues of juvenile triangular bream (*Megalobrama terminalis*) fed the test diets for 8 weeks.

Diet	Whole body	Vertebra	Dorsal muscle
Se0	1.92 ± 0.34^a^	2.21 ± 0.07^a^	0.36 ± 0.07^a^
Se1	3.94 ± 0.33^a^	4.09 ± 0.13^a^	1.20 ± 0.13^b^
Se3	8.35 ± 0.95^b^	10.47 ± 0.18^b^	3.11 ± 0.18^c^
Se9	22.61 ± 1.10^c^	18.53 ± 1.19^c^	8.06 ± 0.24^d^
Se12	28.32 ± 0.94^d^	19.63 ± 1.23^c^	9.43 ± 0.12^e^

Data are presented as mean ± SD (*n* = 3). The data with different superscripts in the same row mean significant difference at *P* < 0.05.

**Table 5:** Effects of selenium yeast on activities of SOD, GSH-Px, and LZM and content of MDA in the liver and kidney of juvenile triangular bream (*Megalobrama terminalis*) fed the test diets for 8 weeks.

Diet	SOD (U/mg prot)		GSH-Px (U/mg prot)		LZM (U/mg prot)		MDA (nmol/mg prot)	
	Liver	Kidney	Liver	Kidney	Liver	Kidney	Liver	Kidney
Se0	551.4 ± 32.6^b^	317.3 ± 7.3^a^	289.9 ± 13.9^a^	363.9 ± 8.9^b^	62.7 ± 2.0^b^	146.8 ± 6.3^b^	4.18 ± 0.03^c^	17.7 ± 0.3^c^
Se1	605.9 ± 5.1^cd^	449.5 ± 9.6^b^	320.0 ± 11.5^b^	432.8 ± 16.9^c^	64.8 ± 1.5^b^	151.7 ± 3.1^b^	3.29 ± 0.07^b^	15.3 ± 0.2^b^
Se3	622.5 ± 5.9^d^	457.2 ± 11.5^b^	429.4 ± 3.2^c^	457.2 ± 11.5^c^	77.7 ± 2.2^c^	192.7 ± 2.7^c^	2.71 ± 0.09^a^	13.9 ± 0.3^a^
Se9	561.8 ± 13.2^bc^	310.2 ± 9.4^a^	329.1 ± 3.9^b^	310.2 ± 9.4^a^	74.5 ± 5.9^c^	119.7 ± 4.2^a^	3.98 ± 0.08^c^	19.5 ± 0.1^d^
Se12	493.9 ± 9.9^a^	321.1 ± 6.1^a^	293.8 ± 6.8^a^	321.1 ± 6.1^a^	53.0 ± 1.2^a^	123.6 ± 2.8^a^	8.85 ± 0.18^d^	19.4 ± 0.4^d^

Data are presented as mean ± SD (*n* = 3). The data with different superscripts in the same row mean significant difference at *P* < 0.05.

## Data Availability

The data that support the findings of this study appear in the submitted article.
